# Correlation of Cardiometabolic Index With Premature Coronary Heart Disease and Coronary Artery Disease Severity in Menopausal Women

**DOI:** 10.31083/RCM48199

**Published:** 2026-06-28

**Authors:** Xiang Sha, Wei Wang, Jian Wang, Jie Qiu, Jianmin Li

**Affiliations:** ^1^Department of Cardiology, The Affiliated Taizhou People's Hospital of Nanjing Medical University, Taizhou School of Clinical Medicine, Nanjing Medical University, 225300 Taizhou, Jiangsu, China

**Keywords:** cardiometabolic, premature, coronary disease, menopause

## Abstract

**Background:**

Premature coronary heart disease (PCHD) is a significant health concern. Therefore, this study aimed to examine the association between the cardiometabolic index (CMI) and PCHD, as well as the severity of coronary artery disease, in menopausal women.

**Methods:**

We conducted a retrospective case-control study including 545 menopausal patients who underwent coronary angiography (CAG) between January 2022 and December 2024. Based on the CAG results, patients were classified into the PCHD and control groups. CMI levels were stratified according to quartiles. Logistic regression models and restricted cubic spline regression were used to examine the relationship between CMI levels and PCHD. CHD severity was assessed using the Gensini score (GS). The association between CMI levels and GS was evaluated using Pearson's correlation and linear regression.

**Results:**

CMI was significantly and positively associated with PCHD in menopausal women (odds ratio (OR) = 1.21, 95% confidence interval (CI) = 1.07–1.36). After adjustment for several potential confounding factors, patients in the highest CMI quartile (Q4) had a higher risk of PCHD than those in the lowest quartile (Q1) (OR = 1.56, 95% CI = 1.05–2.30). There was a linear dose-response relationship between CMI and PCHD risk (*p*_overall_ = 0.003, *p*_non-linear_ = 0.106). Among menopausal patients with PCHD, CMI was positively associated with GS (r = 0.44, *p* < 0.001), and GS in the Q4 group was, on average, 20.94 points higher than that in Q1 reference group.

**Conclusions:**

In menopausal women, CMI is an independent risk factor for PCHD, and elevated CMI levels are significantly correlated with the severity of coronary artery disease.

## 1. Introduction

Cardiovascular diseases are the leading cause of death globally, with the majority of deaths due to stroke and coronary heart disease (CHD) [1]. Premature coronary heart disease (PCHD) is defined as CHD occurring in women under 65 years of age and represents a particularly severe clinical entity. It is frequently associated with acute coronary syndromes, a worse prognosis, and a higher disease burden [2]. PCHD is related to various risk factors, including hypertension, hyperlipidemia, diabetes, smoking, and obesity [3].

Menopausal women constitute a high-risk subgroup for CHD. The decline in endogenous estrogen levels weakens its cardioprotective effects [4], while metabolic changes often lead to a redistribution of body fat from subcutaneous to visceral depots [5]. This shift promotes central obesity and metabolic syndrome, thereby accelerating atherosclerosis [6]. In view of this increased risk, a 2019 global epidemiological survey reported a higher prevalence of PCHD cases among women (2.77 million) than men (2.57 million) [7]. Therefore, early identification of risk in this population is critical for clinical care.

Cardiometabolic index (CMI) quantifies both the distribution of body fat and the degree of abnormal lipid metabolism and is a novel evaluation index for visceral obesity [8]. One study found a close association between CMI and metabolic diseases, including diabetes mellitus, cardiovascular disease, and stroke [9]. However, studies on the relationship between CMI and PCHD, as well as the severity of coronary artery disease in menopausal women, have not been reported. This study aims to investigate these issues through a retrospective case-control study and to explore whether CMI continues to influence PCHD after correction for traditional risk factors. The findings may provide CMI as a potential biomarker for early identification and risk stratification of PCHD in menopausal women, thereby informing future prevention and treatment strategies.

## 2. Materials and Methods

### 2.1 Study Design and Patients

For this retrospective case-control study, we consecutively enrolled postmenopausal female patients younger than 65 years who first underwent diagnostic coronary angiography (CAG) for chest pain or discomfort at the Department of Cardiology of Taizhou People's Hospital of Nanjing Medical University between January 2022 and December 2024. In this study, PCHD is defined as CHD occurring in female patients under the age of 65. CHD was diagnosed when CAG demonstrated ≥50% luminal stenosis in any major coronary artery. Based on the CAG results, patients were divided into the control (non-CHD) and PCHD groups. Patients in both groups were excluded if they had a history of prior percutaneous coronary intervention (PCI) or coronary artery bypass grafting, severe liver dysfunction or renal failure, infectious diseases, malignant tumors, autoimmune diseases, current or recent (within 6 months) hormone replacement therapy, or incomplete clinical data.

The Medical Ethics Committee of Taizhou People's Hospital, affiliated with Nanjing Medical University, reviewed and approved this retrospective study (approval number: KY2024-094-01). Informed consent was not required due to the retrospective nature of the investigation.

### 2.2 Clinical Data Collection

The clinical information collected from patients included age, age at menarche, age at childbearing, age at menopause, height, weight, waist circumference, hip circumference, estradiol (E_2_), leukocytes, neutrophils, hemoglobin, fasting blood glucose, triglycerides (TG), total cholesterol (TC), high-density lipoprotein cholesterol (HDL-C), low-density lipoprotein cholesterol (LDL-C), apolipoprotein A1 (ApoA1), apolipoprotein B (ApoB), lipoprotein (a) (Lp(a)), systolic blood pressure, diastolic blood pressure, hypertension, diabetes mellitus, history of smoking, gravidity, parity, hot flashes, modified Kupperman score and Gensini score (GS). WHtR = waist circumference/height, CMI was calculated as (TG/HDL-C) × WHtR.

### 2.3 CAG and Gensini Score

All patients were adequately assessed and underwent CAG using the standard Judkin technique with multiple positional projections to demonstrate the various coronary vessels. At least one of the main coronary artery branches (left main coronary artery, left anterior descending artery, circumflex artery, and right coronary artery) showing a stenosis degree of ≥50% can be diagnosed as CHD. The severity of coronary artery lesions was then quantified using the GS [10]. GS integrates both the degree of stenosis and the lesion's anatomical location. Specifically, a stenosis score (1 for <25%, 2 for 25–50%, 4 for 51–75%, 8 for 76–90%, 16 for 91–99%, and 32 for total occlusion) is multiplied by a predefined weighting factor corresponding to the functional importance of the specific coronary segment (e.g., 5 for the left main coronary artery, 2.5 for the proximal left anterior descending artery). The total Gensini score for a patient is the sum of these products across all diseased segments, with a higher score indicating more severe coronary disease. All CAG results were interpreted by two interventional cardiologists.

### 2.4 Data Analysis

Continuous variables were presented as mean ± standard deviation or median and quartiles, depending on their distribution. Group comparisons were performed by using the Student's *t*-test or the Mann-Whitney U test, as appropriate. To analyze the association between CMI and PCHD, CMI levels were categorized into quartiles. Adjusted odds ratios (ORs) with 95% confidence intervals (CIs) were derived from logistic regression models that controlled for potential confounders. The nonlinear relationship between CMI and PCHD risk was examined using restricted cubic splines. The correlation between CMI and GS was assessed by Pearson correlation analysis, and their relationship was quantified using linear regression, reporting regression coefficients (β) with 95% CIs. All analyses were performed using R version 4.1.3(R Core Team, Vienna, Austria), with *p *≤ 0.05 considered statistically significant.

## 3. Result

### 3.1 Baseline Characteristics

**Fig. 1.**
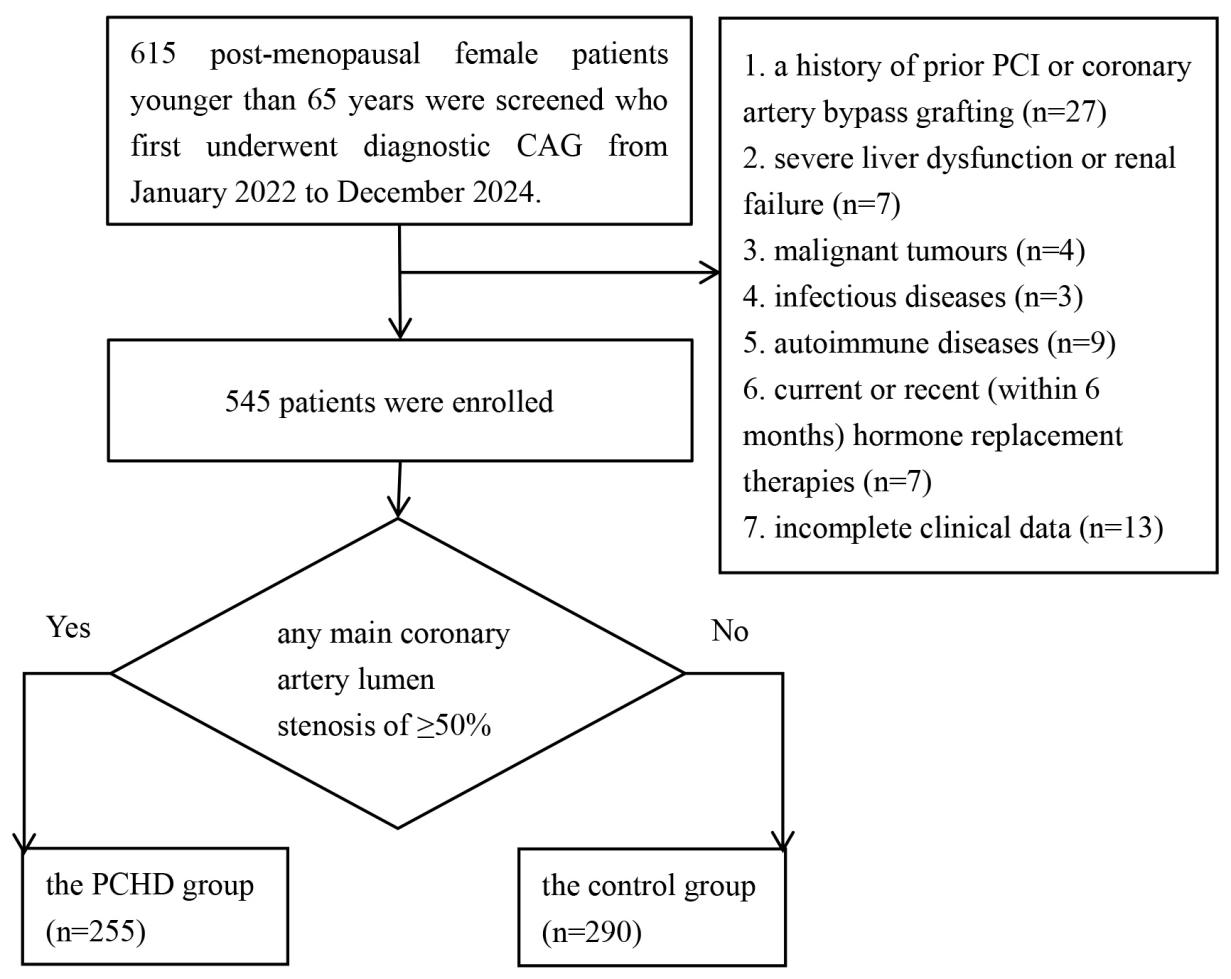
**Flow diagram for patient selection**. CAG, coronary angiography; PCI, percutaneous coronary intervention; PCHD, premature coronary heart disease.

A total of 615 postmenopausal patients were screened, and 545 patients were enrolled in this study (Fig. 1). Based on the CAG results, 255 patients were assigned to the PCHD group, and the remaining 290 patients to the control group.

The baseline characteristics are summarized in Table 1. Compared with the control group, patients in the PCHD group were more likely to have a larger waist circumference, a higher waist-to-hip ratio, a higher proportion of smokers, lower hemoglobin levels, higher TC levels, lower HDL-C levels, and lower ApoA1 levels. A significant difference was found in the modified Kupperman scores between the two groups.

**Table 1.** **Baseline characteristics of patients**.

	Level	Control (n = 290)	PCHD (n = 255)	*p*
Age, years		54.53 (3.59)	54.67 (3.54)	0.658
Height, cm		159.62 (4.59)	159.43 (4.62)	0.624
Age at menopause, years		50.44 (2.32)	50.32 (2.27)	0.519
Age at menarche, years		14.45 (1.75)	14.56 (1.61)	0.454
Age at childbearing, years		24.84 (2.70)	25.15 (2.84)	0.201
Annual income, yuan	<30,000	37 (12.8)	33 (12.9)	0.999
	30,000–80,000	97 (33.4)	85 (33.3)	
	80,000–300,000	120 (41.4)	105 (41.2)	
	>300,000	36 (12.4)	32 (12.5)	
History of miscarriage		139 (47.9)	118 (46.3)	0.764
Parity	1	229 (79.0)	202 (79.2)	0.999
	≥2	61 (21.0)	53 (20.8)	
Gravidity	1	117 (40.3)	104 (40.8)	0.629
	2	79 (27.2)	77 (30.2)	
	≥3	94 (32.4)	74 (29.0)	
Presence of hot flushes		159 (54.8)	131 (51.4)	0.471
Physical exercise	None	121 (41.7)	106 (41.6)	0.986
	Occasionally	79 (27.2)	71 (27.8)	
	Often	90 (31.0)	78 (30.6)	
Waist circumference, cm		83.53 (10.22)	85.70 (9.45)	0.011
Hip circumference, cm		96.31 (6.11)	96.48 (5.65)	0.734
Waist-to-hip ratio		0.87 (0.07)	0.88 (0.07)	0.006
Smoking		35 (12.1)	52 (20.4)	0.011
Hypertension		153 (52.8)	138 (54.1)	0.817
Diabetes		0.09 (0.29)	0.11 (0.32)	0.353
E_2_, pg/mL		23.09 (9.85)	23.34 (9.51)	0.763
WBC, 10^9^/L		6.42 (3.94)	6.12 (3.98)	0.383
Neutrophil, 10^9^/L		3.55 (3.83)	3.64 (4.02)	0.804
Hemoglobin, g/L		131.17 (16.83)	122.27 (17.70)	<0.001
Fasting blood glucose, mmol/L		5.75 (2.08)	5.77 (1.67)	0.922
TG, mmol/L		1.59 (1.11)	1.72 (1.39)	0.205
TC, mmol/L		4.51 (0.95)	5.07 (0.98)	<0.001
Modified Kupperman score	Normal	7 (2.4)	4 (1.6)	0.014
	Mild	62 (21.4)	39 (15.3)	
	Moderate	176 (60.7)	146 (57.3)	
	Severe	45 (15.5)	66 (25.9)	
HDL-C, mmol/L		1.30 (0.30)	1.01 (0.29)	<0.001
LDL-C, mmol/L		2.52 (0.71)	2.55 (0.73)	0.565
ApoA1, g/L		1.18 (0.25)	1.01 (0.21)	<0.001
ApoB, g/L		0.81 (0.19)	0.81 (0.20)	0.737
LP(a), mg/L		112.00 [52.25, 273.00]	125.00 [64.50, 329.50]	0.163

Note: Monetary values were converted using the average exchange rate during the patient enrollment period from 2022 to 2024: 1 Chinese yuan (CNY) = 0.1436 US dollars (USD). Values are mean (SD), median [Q1, Q3], or n (%). E_2_, estradiol; WBC, white blood cell; TG, triglycerides; TC, total cholesterol; HDL-C, high-density lipoprotein cholesterol; LDL-C, low-density lipoprotein cholesterol; ApoA1, apolipoprotein A1; ApoB, apolipoprotein B; LP(a), lipoprotein (a).

### 3.2 Relationship Between CMI and PCHD

In the unadjusted model, CMI was significantly and positively correlated with PCHD (OR = 1.21, 95% CI = 1.07–1.36) (Table 2). After adjusting for confounding factors, a significant positive association between CMI and PCHD remained. Patients were divided into four groups according to CMI quartiles: the first quartile (Q1) group (≤0.27), second quartile (Q2) group (0.27–0.60), third quartile (Q3) group (0.60–0.96), and fourth quartile (Q4) group (≥0.96). After adjustment for age, age at menarche, age at childbearing, age at menopause, annual income, waist-to-hip ratio, smoking, hemoglobin, TC, modified Kupperman score, HDL-C, and ApoA1, the odds of PCHD were significantly higher in the Q4 group compared to the Q1 group (OR = 1.56, 95% CI = 1.05–2.30). The risk of PCHD increased with higher CMI levels (*p* = 0.009).

**Table 2.** **Logistic regression analyses for the association of CMI and PCHD**.

CMI	Model 1	Model 2	Model 3
Continuous#	1.21 (1.07–1.36)	1.24 (1.08–1.42)	1.23 (1.07–1.41)
Q1 (≤0.27)	Ref	Ref	Ref
Q2 (0.27–0.60)	0.98 (0.70–1.38)	1.08 (0.74–1.58)	1.05 (0.71–1.55)
Q3 (0.60–0.96)	1.53 (1.09–2.15)	1.48 (1.01–2.17)	1.43 (0.97–2.11)
Q4 (≥0.96)	1.53 (1.09–2.15)	1.61 (1.10–2.37)	1.56 (1.05–2.30)
*p* for trend	0.002	0.005	0.009

Note: Model 1 was a crude model. Model 2 was adjusted for waist-to-hip ratio, smoking, hemoglobin, TC, modified Kupperman score, HDL-C, and ApoA1. Model 3 was additionally adjusted for age, age at menarche, age at childbearing, age at menopause, and annual income, in addition to Model 2. CMI, cardiometabolic index. #The effect per 1 SD increase in CMI.

**Fig. 2.**
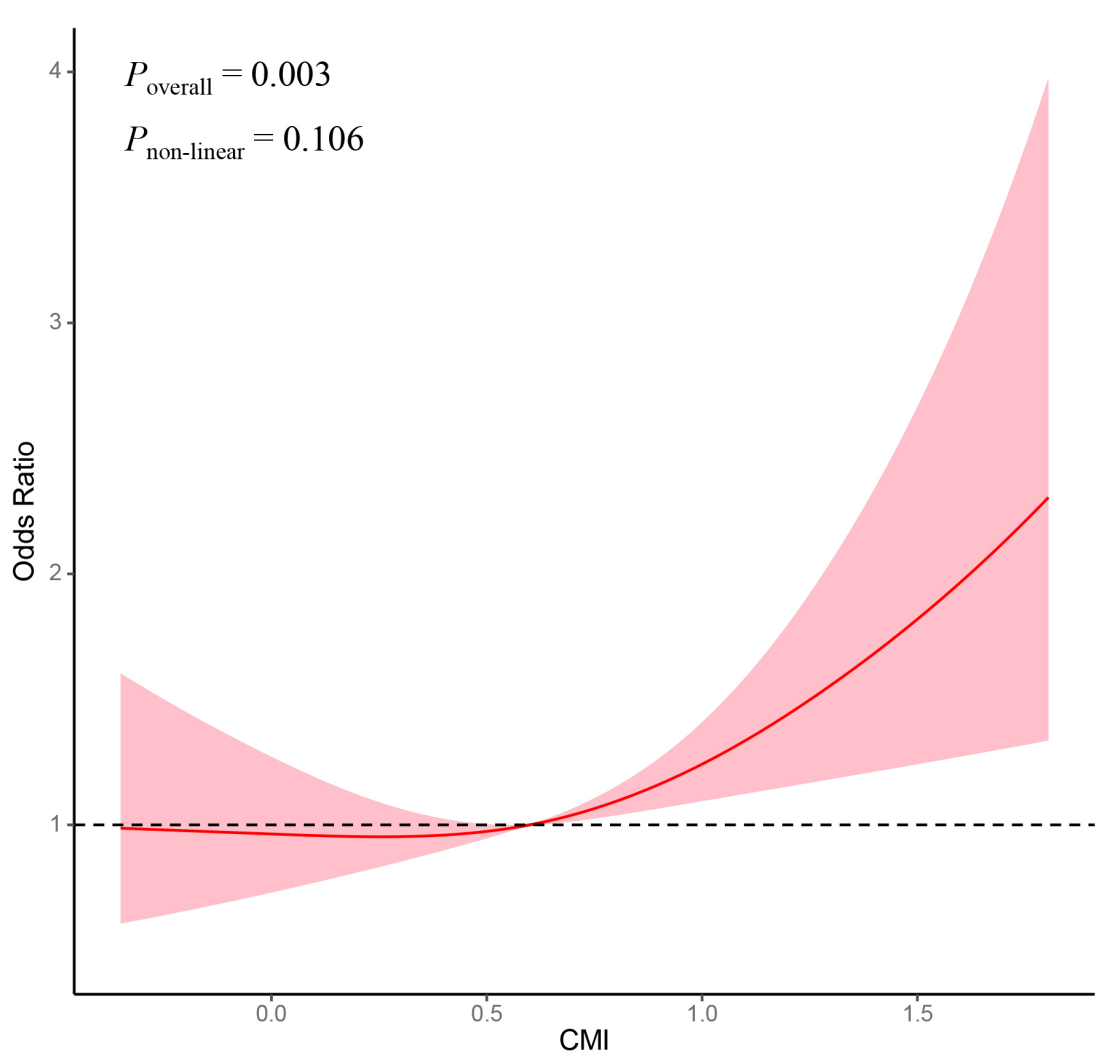
**Cubic spline analyses for the association between CMI and the risk of PCHD**.

The restricted cubic spline curve showed that, after adjustment for age, age at menarche, age at childbearing, age at menopause, annual income, waist-to-hip ratio, smoking, hemoglobin, TC, modified Kupperman score, HDL-C, and ApoA1, the risk of PCHD in menopausal patients gradually increased with increasing CMI level. There existed a linear dose–response relationship between CMI and the risk of PCHD (*p*_overall_ = 0.003, *p*_non-linear_ = 0.106) (Fig. 2).

### 3.3 Relationship Between CMI and GS

**Fig. 3.**
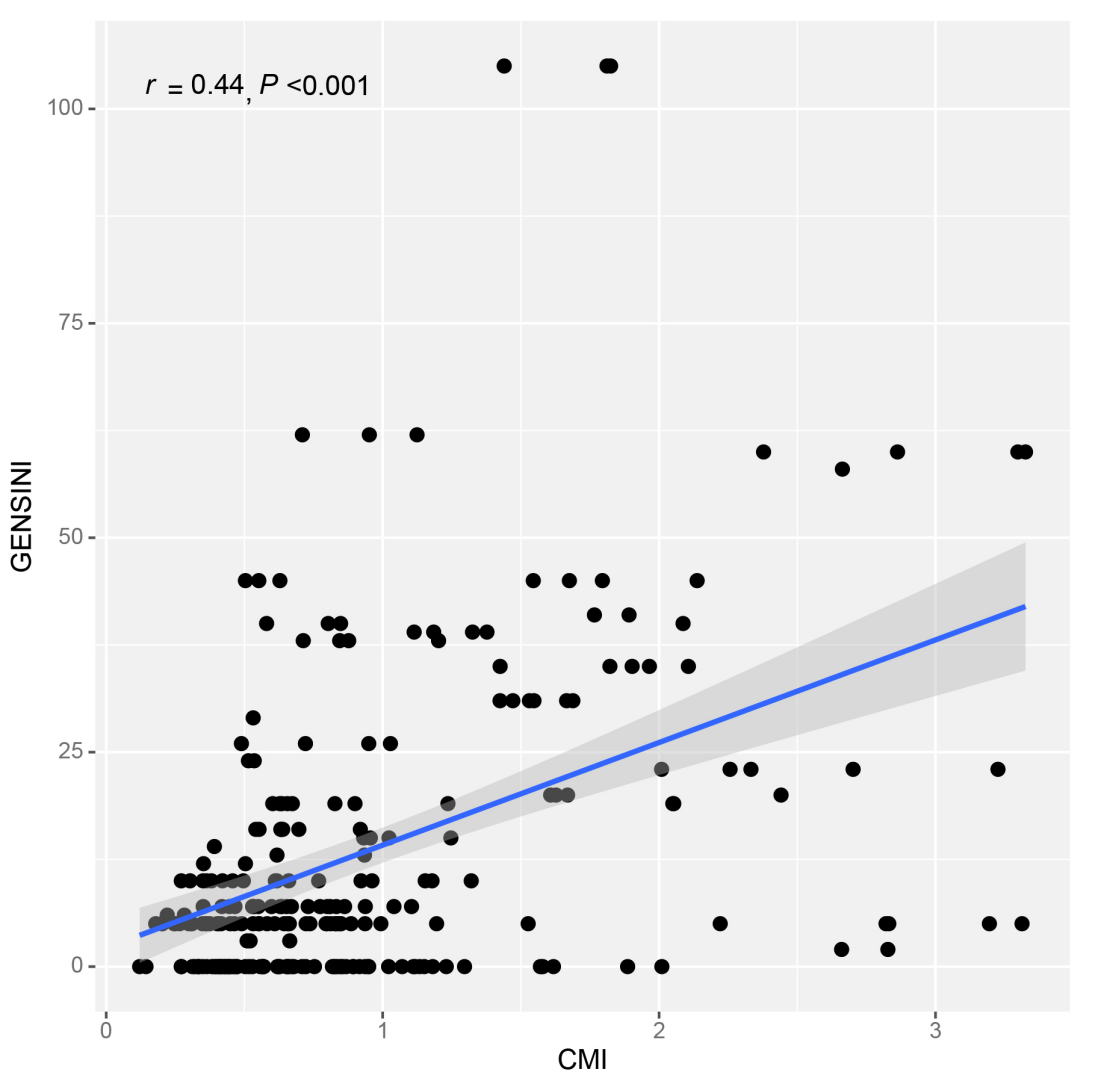
**The association between CMI and GS in PCHD patients**.

Among menopausal patients with PCHD, there was a positive association between CMI and GS (r = 0.44, *p* < 0.001) (Fig. 3).

To further quantify the association between CMI and coronary artery disease severity in PCHD patients, linear regression analyses were performed. In the unadjusted model, CMI was significantly associated with higher GS (β = 8.08, 95% CI = 6.05–10.12) (Table 3). This association persisted after adjustment for several confounders. When analyzed by CMI quartiles, the GS of patients in the Q4 group was on average 20.94 points higher than that of the Q1 reference group (95% CI = 14.75–27.14) after adjustment for age, age at menarche, age at childbearing, age at menopause, annual income, waist-to-hip ratio, smoking, hemoglobin, TC, modified Kupperman score, HDL-C, and ApoA1.

**Table 3.** **Linear regression analyses for the association of CMI and GS in PCHD patients**.

CMI	Model 1	Model 2	Model 3
Continuous#	8.08 (6.05–10.12)	7.38 (5.31–9.45)	7.10 (5.01–9.19)
Q1 (≤0.27)	Ref	Ref	Ref
Q2 (0.27–0.60)	4.70 (–1.49–10.88)	3.60 (–2.22–9.42)	3.58 (–2.33–9.49)
Q3 (0.60–0.96)	6.47 (0.21–12.74)	4.35 (–1.61–10.31)	4.24 (–1.77–10.24)
Q4 (≥0.96)	22.64 (16.39–28.89)	21.71 (15.59–27.83)	20.94 (14.75–27.14)

Note: Model 1 was a crude model. Model 2 was adjusted for waist-to-hip ratio, smoking, hemoglobin, TC, modified Kupperman score, HDL-C, and ApoA1. Model 3 was additionally adjusted for age, age at menarche, age at childbearing, age at menopause, and annual income, in addition to Model 2. #The effect per 1 SD increase in CMI.

## 4. Discussion

In the present study, we discovered a significant positive correlation between CMI and PCHD risk. The cubic spline analysis indicated that the relationship between CMI and PCHD followed a linear trend, providing clear evidence that CMI may play a crucial role in assessing PCHD risk in menopausal women. CMI was positively associated with coronary artery disease severity. Overall, as an emerging assessment tool, CMI could effectively reflect the potential risks and severity of cardiovascular diseases in menopausal women.

PCHD represents a unique clinical type of CHD, characterized by an acute onset and atypical symptoms, leading to significant impacts on the quality and lifespan of affected individuals [7]. PCHD patients are at risk for acute coronary syndrome upon plaque rupture and thrombosis, although many may remain in a stable ischemic state for prolonged periods. It is imperative to note that in postmenopausal patients presenting with chest pain, careful consideration must be given to differential diagnoses such as Takotsubo syndrome and spontaneous coronary artery dissection, which are relatively common in this age group. In this study, a precise diagnosis of CHD was made using CAG, which helps distinguish it from other causes. The etiology of PCHD is multifactorial, making early identification of risk factors crucial for effective prevention, diagnosis, and treatment, ultimately reducing morbidity and mortality rates. Recent studies have shown a higher incidence of CHD in menopausal women compared to premenopausal women [11]. Moreover, the prevalence of visceral obesity, another critical risk factor for CHD, is also significantly higher in postmenopausal women [12]. Considering these findings, this study explores for the first time the relationship between CMI and the onset and severity of PCHD, primarily focusing on menopausal women.

Visceral obesity is an independent risk factor for coronary atherosclerosis and is known to exacerbate the severity of CHD, negatively influencing patients' prognoses [13]. A study using data from the Multi-Ethnic Study of Atherosclerosis found a strong association between visceral fat, as measured by abdominal computed tomography, and coronary artery calcification (CAC). A greater area and higher density of abdominal visceral fat were associated with an increased likelihood of CAC [14]. Furthermore, visceral to subcutaneous adipose tissue ratio has been confirmed as an independent risk factor for CHD severity [15]. CMI combines TG/HDL-C, which reflects dyslipidemia, and WHtR, which reflects the degree of obesity. It is an indicator that reflects both the degree of obesity and lipid levels and can be used to evaluate visceral obesity [16]. Moreover, numerous studies have found that CMI is strongly associated with hypertension, congestive heart failure, and cardiovascular disease [17]. In our research, we found a significant positive correlation between CMI and the risk of PCHD. Furthermore, CMI was positively correlated with the severity of coronary artery lesions in PCHD patients. To the best of our knowledge, this is the first study to highlight the independent predictive value of CMI for PCHD and its association with CHD severity in menopausal women.

The decline in estrogen levels following menopause leads to abnormal lipid metabolism and a redistribution of visceral fat [18], potentially serving as a key mechanism underlying the enhanced association between CMI and PCHD. Postmenopausal women tend to develop visceral obesity and excess intra-abdominal fat, which elevates cardiometabolic risk [19]. Visceral fat acts as a highly active endocrine organ, secreting large amounts of free fatty acids (FFA), which are transported directly to the liver through the portal vein. This process promotes excessive secretion of very low-density lipoproteins, contributing to the progression of atherosclerosis [20]. Furthermore, visceral adipose tissue secretes pro-inflammatory adipokines that contribute to inflammation and insulin resistance [21]. Insulin resistance leads to increased breakdown of fat tissue, further releasing FFAs, adipokines, and inflammatory factors into the bloodstream, creating a vicious cycle. These metabolic and inflammatory disorders damage the vascular endothelium, which serves as the primary trigger for CHD onset [22]. Therefore, CMI not only reflects fat distribution but also interacts with factors such as inflammation and metabolic disorders, which can contribute to the onset and progression of cardiovascular disease. Beyond its strong predictive power for PCHD incidence, CMI also serves as a direct indicator of cumulative coronary artery damage resulting from the interplay of these harmful factors, making it a valuable tool for precisely assessing the extent and severity of coronary injury and providing insights for early clinical intervention.

In clinical practice, the identification of elevated CMI could assist in risk stratification for postmenopausal women, allowing for targeted prevention strategies. These could include interventions to reduce visceral fat accumulation, address metabolic abnormalities, and manage inflammatory responses. Future research should focus on applying CMI in longitudinal studies to better understand its role in predicting PCHD progression and informing treatment protocols.

## 5. Strengths and Limitations

This study's strength lies in the application of the CMI, an emerging and comprehensive tool for assessing metabolic abnormalities. This tool holds promise for clinical application in menopausal women, a specific population that may benefit significantly from such assessments. However, some limitations must be acknowledged. Firstly, due to the study's retrospective design, lifestyle factors and family history of CHD in first-degree relatives were not comprehensively recorded, both of which could serve as potential risk factors for PCHD. Furthermore, the possibility of other unmeasured confounding cannot be excluded. Secondly, as the aim was to investigate the association between CMI and PCHD, our analysis did not perform subgroup comparisons based on clinical subtypes of CHD. Future research should investigate whether this association varies across clinical phenotypes. Thirdly, the use of a self-selected sample may limit the generalizability of our findings to the broader population. Finally, as a retrospective study, causal relationships cannot be conclusively established.

## 6. Conclusions

In conclusion, this study reveals a positive correlation between CMI and the risk of PCHD, as well as the severity of coronary artery lesions in menopausal women, further supporting the potential of CMI as an effective tool for assessing cardiovascular disease risk. The findings offer valuable insights and highlight the importance of early identification and prompt intervention in cardiovascular diseases among menopausal women. Overall, these results provide a solid foundation for future research on early screening and personalized therapeutic strategies for cardiovascular diseases.

## Data Availability

The original contributions presented in the study are included in the article. Further inquiries can be directed to the corresponding author.
